# Rapamycin Resistant Murine Th9 Cells Have a Stable In Vivo Phenotype and Inhibit Graft-Versus-Host Reactivity

**DOI:** 10.1371/journal.pone.0072305

**Published:** 2013-08-21

**Authors:** Courtney W. Mangus, Paul R. Massey, Daniel H. Fowler, Shoba Amarnath

**Affiliations:** 1 Experimental Transplantation and Immunology Branch, National Cancer Institute, National Institutes of Health, Bethesda, Maryland, United States of America; 2 Howard Hughes Medical Institute, National Institute of Health Research Scholars Program, Chevy Chase, Maryland, United States of America; International Center for Genetic Engineering and Biotechnology, India

## Abstract

The cytokine micro-environment can direct murine CD4^+^ T cells towards various differentiation lineages such as Th1, Th2 and Tregs even in the presence of rapamycin, which results in T cells that mediate increased in vivo effects. Recently, a new lineage of T cells known as Th9 cells that secrete increased IL-9 have been described. However, it is not known whether Th9 differentiation occurs in the presence of rapamycin or whether adoptively transferred donor Th9 cells would augment or restrict alloreactivity after experimental bone marrow transplantation. We found that CD4^+^ T cells that were co-stimulated and polarized with TGF-β and IL-4 in the presence or absence of rapamycin each yielded effector cells of Th9 phenotype that secreted increased IL-9 and expressed a transcription factor profile characteristic of both Th9 and Th2 cells (high GATA-3/low T-bet). Augmentation of T cell replete allografts with manufactured rapamycin resistant Th9 cells markedly reduced both CD4^+^ and CD8^+^ T cell engraftment and strongly inhibited allo-specific T cell secretion of IFN-γ. The potency of Th9 cell inhibition of alloreactivity was similar to that of rapamycin resistant Th2 cells. Importantly, rapamycin resistant Th9 cells persisted and maintained their cytokine phenotype, thereby indicating limited differentiation plasticity of the Th9 subset. As such, Th9 differentiation proceeds in the presence of rapamycin to generate a cell therapy product that maintains high IL-9 expression in vivo while inhibiting IFN-γ driven alloreactivity.

## Introduction

CD4^+^ T helper subsets can differentiate into various lineages in response to environmental cues such as cytokines or ligand interactions. Of the various lineages of T helper subsets, Th9 cells are a recent addition. Th9 cells develop from CD4 precursors in response to TGF-β and IL-4 and are primarily characterized by their increased secretion of IL-9 [1]. While IL-9 producing CD4^+^ T cells were previously classified as Th2 cells [2], recent studies have shown that Th9 cells are a distinct lineage of T helper cells [1], [3]. Th9 cells have been implicated in various disease processes that have been associated with Th2 cells, including: helminth infection [4] [1], allergy [5] and asthma [6] [7]. However, Th9 cells can share mechanistic features of Th1 cells, as they have been associated with solid organ graft rejection [8], experimental peripheral neuritis [3] and anti-tumor responses [9]. On the other hand, the tolerogenic properties of Th9 cells were noted in a transplantation setting where IL-9 promoted allograft tolerance [10]. As such, there are somewhat conflicting data with respect to the role of Th9 cells in immunity, and no data exists concerning their potential role after experimental allogeneic bone marrow transplantation.

Rapamycin is an immunosuppressive drug that blocks cell surface signaling through inhibition of the kinase, mammalian target of rapamycin (mTOR) [11]. Although rapamycin can preferentially expand regulatory T cells (Tregs) [12] [13] in some models, we have shown that polarization towards Type I and Type II CD4^+^ T cells [14] [15], [16], [17] can be achieved in the presence of rapamycin provided that APC free co-stimulation is administered along with sufficient polarizing cytokines. In murine transplant models [17], [18] and in a human-into-mouse xenogeneic transplant model [15], we have shown that such rapamycin resistant effector T cell subsets possess an anti-apoptotic phenotype [19], persist in vivo after adoptive transfer, have stability of the defined cytokine phenotype and mediate increased transplantation effects. A phase II clinical trial of rapamycin resistant T cells has been completed [20].

Given the apparent role of Th9 cells in mediating anti-tumor effects [9] while limiting inflammation [10], and the potential beneficial role of ex vivo rapamycin for cell therapy efforts [21], we initiated an effort to determine whether: (1) it might be possible to achieve Th9 polarization in the presence of rapamycin; (2) such rapamycin resistant Th9 cells might maintain their cytokine phenotype in vivo; and (3) in the context of a T cell replete model of experimental bone marrow transplantation, allograft augmentation with Th9 cells might modulate IFN-γ driven graft-versus-host reactivity.

## Materials and Methods

### Animals

BALB/c (H-2K^d^), C57BL/6 (H-2K^b^) and Ly5.1 congenic (H-2K^b^) mice were obtained from Frederick Cancer Research facility (Frederick, MD). All mice were 6–8 weeks old, maintained in a specific pathogen-free facility at the National Institutes of Health. Experiments were performed in accordance with the guidelines of the NIH Animal Care and Use Committee (ACUC). The protocol was approved by the NCI ACUC (protocol number MB-075).

### In vitro Generation of Rapamycin-resistant Th9 Cells

Spleen cells from C57BL/6 mice were harvested and lysed using ACK lysing buffer (Quality Biological, Inc., Gaithersburg, MD). B cells were depleted using goat-anti-mouse conjugated magnetic bioparticles (Polysciences Inc., Warrington, PA). To generate CD3, CD28 mAb coated beads; M450 Dynabeads (Dynal ASA, Oslo, Norway) were incubated with anti-murine CD3 and CD28 mAbs (BD Pharmingen, San Diego, CA) in 0.1M-borate solution at 37°C overnight. CD3, CD28 mAb coated beads were then washed with PBS containing 3% FBS (Gemini Bio-Products, Woodland, CA), 5mM EDTA (Quality Biological, Inc.) and 0.1% sodium azide (Sigma, St. Louis, MO), and brought up in the same solution at a concentration of 40×10^6^ beads/ml. Control rapamycin resistant Th1 cells (Th1.R) were generated using αCD3/αCD28-coated beads in RPMI complete media with 20 IU/mL rhIL-2, 20 ng/mL rhIL-7, 20 ng/ml rmIL-12 (Peprotech), 10 mM of Rapamycin (LC Laboratories, Woburn, MA) and 3.3 mM NAC. Control rapamycin resistant Th2 cells (Th2.R) were cultured exactly the same except for the addition of 1000 IU/ml of rm IL-4 instead of rmIL-12. Culture conditions for the manufacture of Th9 cells were exactly the same as for Th2 cells except for the further addition of 5 ng/ml rhTGF-β1. Specific cytokine-containing complete media was added to all cultures daily from days 2–6 to maintain a cell concentration between 0.2–1.0×10^6^ cells/mL. After 6 days in culture, the various T helper cell subsets were used for bone marrow transplant experiments, multiplex bead array, and flow cytometry analysis.

### Cytokine Secretion Assays

Rapamycin-generated Th2 (Th2.R) and Th9 (Th9.R) cells were harvested (day 6) and adjusted to 1×10^6^ cells/ml in 24-well plates with or without αCD3/αCD28 beads. Twenty-four-hour culture supernatants were collected; cytokine content was evaluated by Bio-Plex multiplex sandwich immunoassay (Bio-Rad).

### Bone Marrow Transplantation

Host BALB/c (H-2K^d^) mice were lethally irradiated (950cGy; ^137^Cs g-radiation source; Gamma Cell 40; Atomic Energy of Canada, Ottawa, Canada) and reconstituted with parental B6 (H-2K^b^) bone marrow cells (5×10^6^ cells) and pan T cells (1×10^6^ cells) intravenously (i.v.). Some cohorts received in vitro cultured Th2.R (10×10^6^ cells) or Th9.R (10×10^6^ cells) cells. In vivo alloreactivity was allowed to occur and immune biology was studied at day 14 post-transplant.

### Flow Cytometry Analysis of T Cells

Splenocytes from BALB/c mice were obtained at day 14 post-BMT. Day 14 splenocytes were stained with CD4 PE-cy5 (H129.19), H_2_K^b^ PE (AF6.88.5), CD3 FITC (145-2C11), CD45.1 APC (A20) and CD45.2 PE-cy7 (104). For intracellular cytokine measurement, T cells were stimulated for 4h with phorbol 12-myristate 13-acetate and ionomycin (Sigma) followed by the addition of Brefeldin A (GolgiPlug) and Monensin (Golgi stop) for the last 2hrs. Cells were then fixed, permeabilized and stained to detect IFN-γ FITC (XMG1.2), IL-9 PE (RM9A4) and IL-4 APC (11B11). All antibody staining was performed in the presence of αCD16/32 (9.3; eBioscience). Cells were then analyzed using LSRII and FlowJo software. All antibodies were obtained from BD Bioscience unless otherwise stated.

### In Vivo Tracking of Donor Th2.R and Th9.R Populations and Unmanipulated CD4 and CD8 T Cells

To allow cell tracking, Th2.R and Th9.R cells were generated using splenic T cells from CD45.1 C57BL/6 congenic mice. Anti-CD45.2 PE.cy7(BD Pharmingen) was used to track unmanipulated CD4 and CD8 populations.

### Determination of in vivo Allosensitization

Spleen cells collected post-BMT were subjected to: no stimulation, αCD3/αCD28 stimulation, B6 syngeneic DC stimulation or BALB/c allogeneic DC stimulation. For αCD3/αCD28 stimulation, spleen cells were adjusted to a concentration of 0.5×10^6^ cells/ml and incubated with CD3/CD28 beads. DCs were obtained by culturing marrow cells for 4 days in rmGM-CSF and rmIL-4 (each at 1000 IU/ml; PeproTech); bacterial LPS (1 µg/ml; Calbiochem) was added to final 24 h of DC culture. Expanded DC were washed and used at a ratio of 10∶1 (spleen cell:DC). For cytokine analysis, twenty-four-hour culture supernatants were collected; cytokine content (IL-4, IL-9, IFN-γ and TNF-α) was evaluated by Bio-Plex multiplex sandwich immunoassay (Bio-Rad).

### Statistics

Statistical analysis was determined using GraphPad Prism 4 software. P values from data presented as histograms, unless stated otherwise, were determined using a two-tailed unpaired *t* test. Unless stated otherwise, histogram columns represent the mean values for each experiment and error bars indicate the standard error of mean. Single asterisk (*) denotes *P<0.05*, double asterisk (**) denotes *P<0.01*, and triple asterisk (***) denotes *P<0.001*.

## Results

### Th9 Polarization Occurs in the Presence of Rapamycin

**Figure 1:**
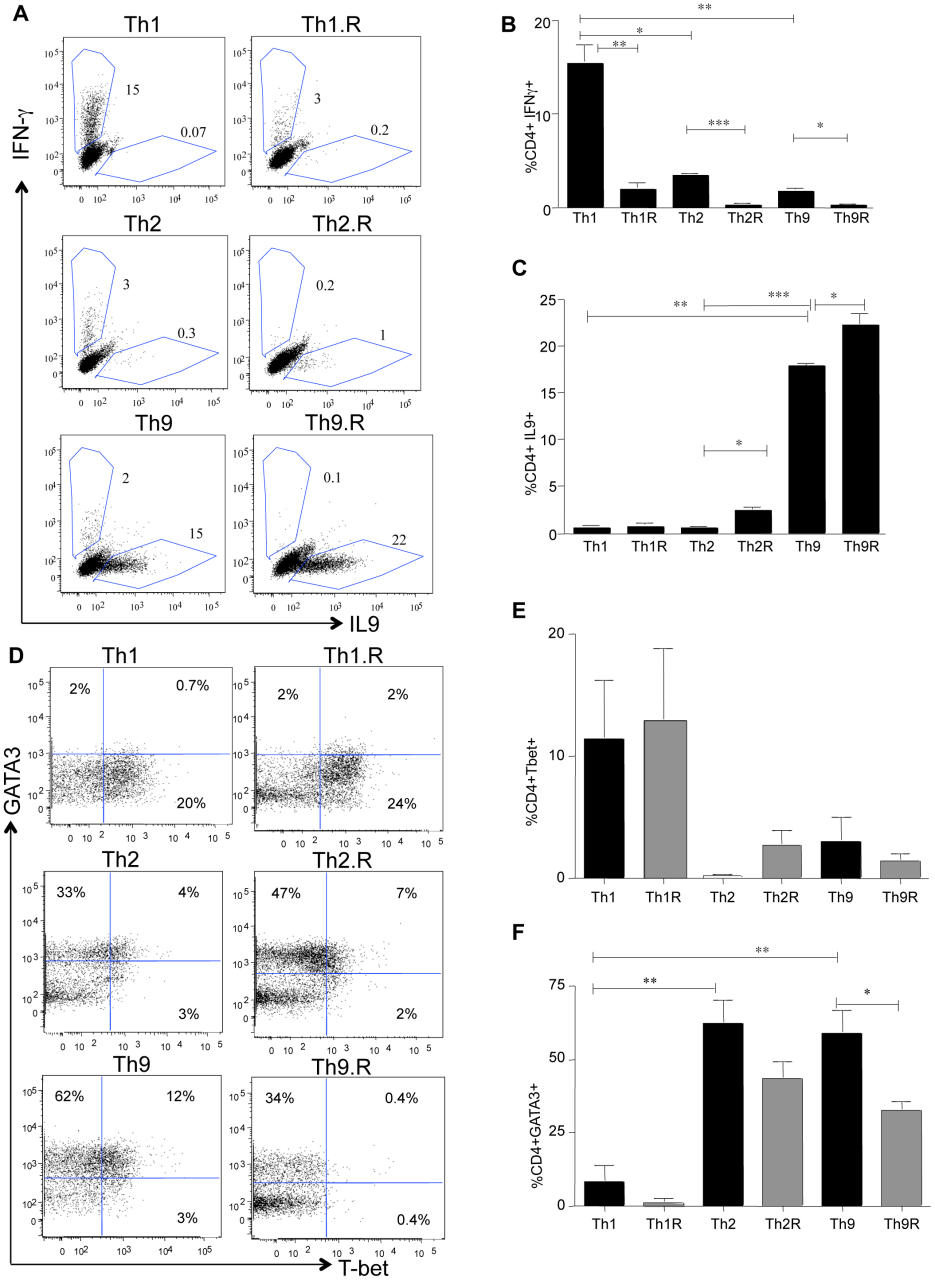
Th9 polarization proceeds in the presence of rapamycin. Splenocytes were harvested from C57B6 mice and bulk CD4^+^ T cells were isolated. CD4^+^ T cells were polarized to either Th1 or Th2 or Th9 differentiation for 6 days. At day 6, cells were washed and characterized for cytokine production and transcription factor expression by I.C. flow cytometry. (A) Representative dot plot showing IL-9 versus IFN-γ production in Th1 cells, Th1 cells grown in rapamycin (Th1.R), Th2 cells, Th2.R, Th9 cells and Th9.R cells. (B) Summary of three independent experiments showing IFN-γproduction in the various CD4^+^ T helper subsets. (C) Summary of three independent experiments showing IL-9 production in the various CD4^+^ T helper subsets. (D) Representative dot plot showing T-bet versus GATA3 expression in Th1 cells, Th1.R cells, Th2 cells, Th2.R, Th9 cells and Th9.R cells. (E) Summary of three independent experiments showing T-bet expression in the various CD4^+^ T helper subsets. (F) Summary of three independent experiments showing GATA3 expression in the various CD4^+^ T helper subsets. Single asterisk (*) denotes *P<0.05*, double asterisk (**) denotes *P<0.01*, and triple asterisk (***) denotes *P<0.001.*

Using intra-cellular cytokine flow cytometry, we found that bulk CD4^+^ T cells were capable of being polarized into Th1, Th2 or Th9 cells in the presence or absence of rapamycin (Representative data; Figure 1a). The frequency of IFN-γ secreting cells was reduced in each subset by rapamycin, with the Th2 and Th9 subsets being lower than the Th1 subset (Figure 1b). Both Th9 and Th9.R cultures had an increased frequency of IL-9 secreting cells relative to the other subsets (Figure 1c). Using transcription factor I.C. flow cytometry, we found that T-bet was highly expressed by Th1 and Th1.R cells (Figure 1d and 1e) whereas both Th2/Th2.R and Th9/Th9.R cells preferentially expressed GATA3 (Figure 1d and 1f).

### Murine Th9 Cells Expanded in Rapamycin Secrete IL-9 in vitro

**Figure 2:**
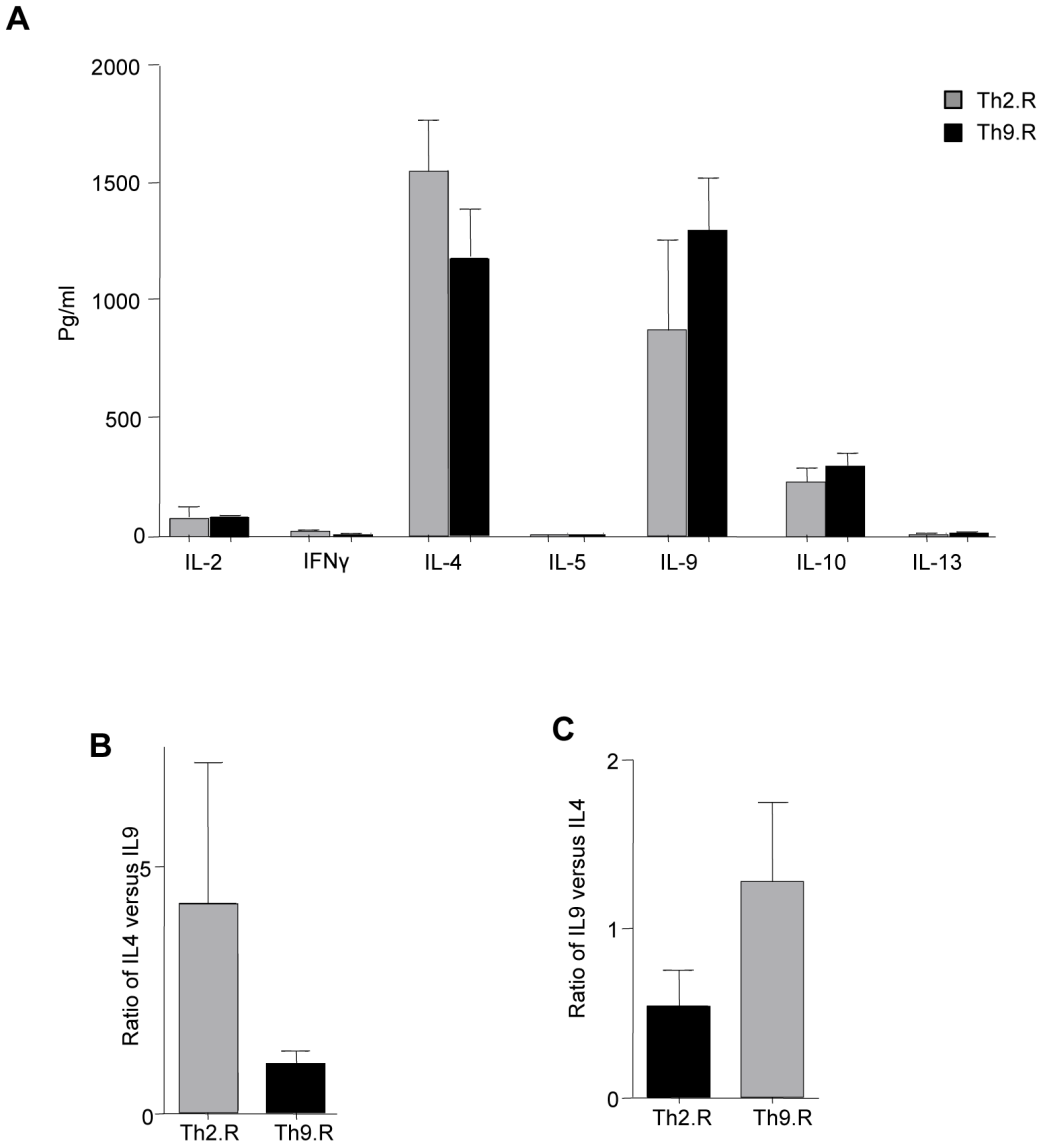
Cytokine Profile of CD4^+^ Th9.R cells. Splenocytes were harvested from C57B6 mice and bulk CD4^+^ T cells were isolated. CD4^+^ T cells were polarized to either Th1 or Th2 or Th9 differentiation for 6 days. At day 6, cells were washed and characterized for cytokine production by Multiplex Luminex assay. (A) Shows Th1/Th2 cytokine secretion profile of Th2 and Th9 cells expanded in the presence of rapamycin. (B) Ratio of IL-4 versus IL-9 cytokine production in Th2.R and Th9.R cells, (C) Ratio of IL-9 versus IL-4 cytokine production in Th2.R and Th9.R cells.

Using multiplex bead array system, the cytokine secretion profile of Th2.R and Th9.R was tested. Th2.R and Th9.R secreted low levels of IL-2 and IFN-γ (Figure 2a) and similar magnitudes of IL-4 and IL-9 (Figure 2a). The ratio of IL-4 to IL-9 tended to be higher in the Th2.R subset but this difference did not reach statistical significance (Figure 2b); similarly, the ratio of IL-9 to IL-4 tended to be higher in the Th9.R subset but did not reach statistical significance (Figure 2c).

### Murine Th9.R Cells Inhibit IFN-γ Mediated GVHR

**Figure 3:**
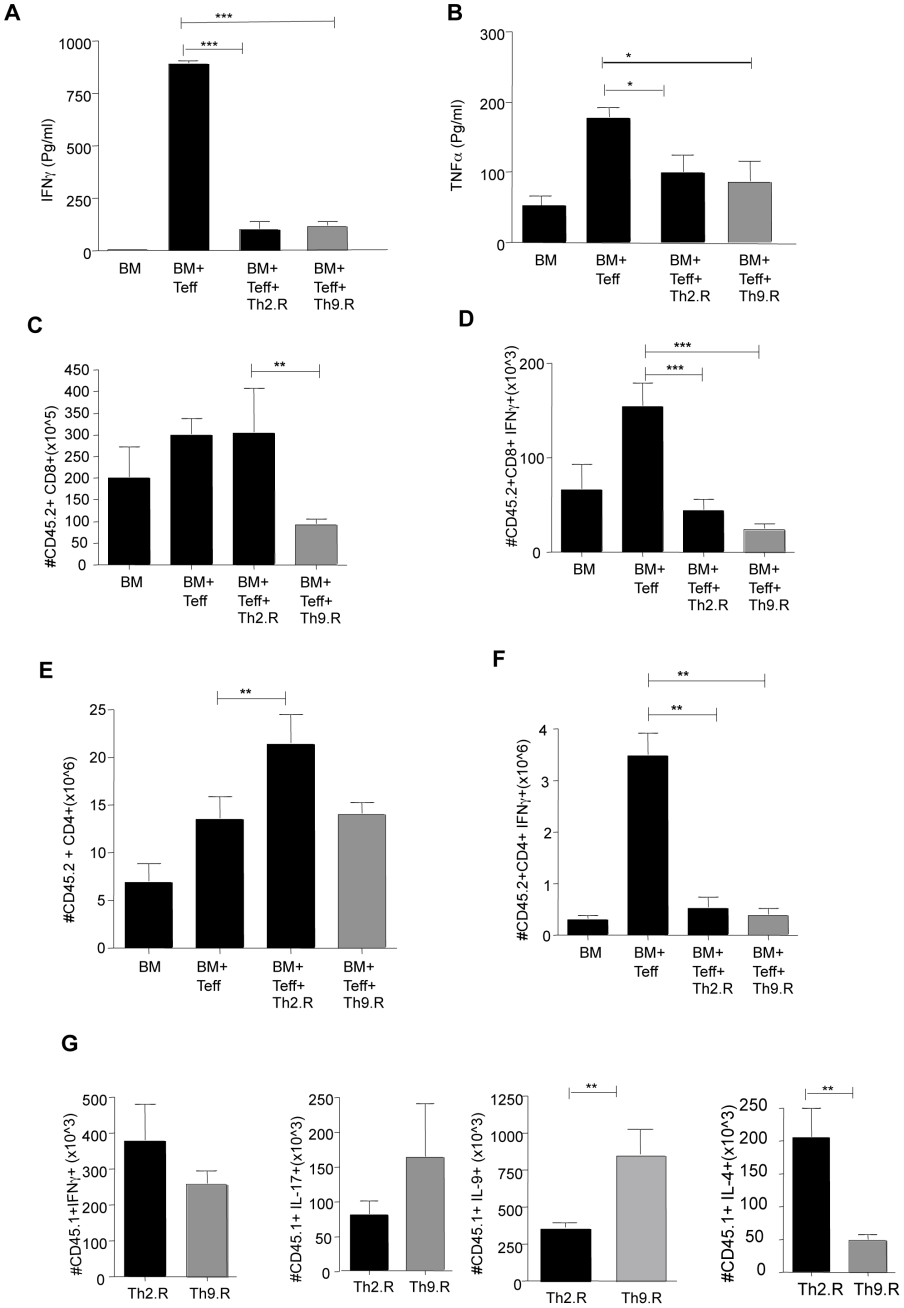
Rapamycin resistant Th9 cells inhibit alloreactivity in vivo. Host BALB/c mice were subjected to TBI (950cGY) and then reconstituted with allogeneic CD45.2 B6 BM (10M) and T cells (1M). In certain cohorts, mice received either Th2.R or Th9.R cells (10M) that were generated from congeneic CD45.1 mice. At day 14, splenocytes were harvested and restimulated with either syngeneic or allogeneic DC. (A) Alloreactive IFN-γ secretion in the different cohorts. (B) Alloreactive TNF-α secretion. (C) Absolute numbers of donor CD45.2^+^ CD8^+^ T cell engraftment in the various cohorts at day 14 post-BMT. (D) Absolute numbers of post-BMT donor CD8^+^ T cells that produce IFN-γ after PMA/ionomycin stimulation was detected by I.C. flow cytometry. (E) Absolute number of donor CD45.2^+^ CD4^+^ T cell engraftment in the various cohorts at day 14 post-BMT. (F) Absolute numbers of post-BMT donor CD45.2^+^ CD4^+^ T cells that produce IFN-γ after PMA/ionomycin stimulation. (G) Ex vivo cytokine production of Th2.R and Th9.R cells at day 14 post BMT. Each experiment had n = 5 animals per cohort. Single asterisk (*) denotes *P<0.05*, double asterisk (**) denotes *P<0.01*, and triple asterisk (***) denotes *P<0.001.*

Murine recipients were reconstituted with BM and allogeneic T cells following TBI. Certain cohorts received additional Th2.R or Th9.R cells that were expanded from CD45.1 C57BL6 donors. At day 14, splenocytes from cohorts that were treated with Th2.R or Th9.R cells secreted reduced IFN-γ and TNF-α in response to allogeneic DC stimulation (Figure 3a, 3b). Co-transfer of Th9.R cells, but not Th2.R cells, resulted in decreased engraftment of donor CD8^+^ T cells (Figure 3c). However, both Th9.R and Th2.R cells reduced the frequency of IFN-γ producing donor CD8^+^ T cells (Figure 3d). By comparison, Th2.R cells but not Th9.R cells increased the number of post-transplant donor CD4^+^ T cells that were derived from the unmanipulated T cell inoculum (CD45.2^+^)(Figure 3e). Both Th9.R and Th2.R cells reduced the frequency of IFN-γ producing donor CD45.2^+^CD4^+^ T cells (Figure 3f). At day 14 post-BMT, the adoptively transferred Th2.R and Th9.R populations were present in vivo at similar levels (absolute number of Th2.R vs. Th9.R cells, ×10^6^ per spleen; Mean ± SEM: 9.3±1.9 vs. 7±1.7). Similar to their phenotype at the time of adoptive cell transfer, Th9.R and Th2.R cells harvested at day 14 post- BMT preferentially produced either IL-9 or IL-4, respectively (Figure 3g).

## Discussion

In this study, we have initiated an evaluation of the in vitro phenotype and in vivo stability of rapamycin resistant Th9 cells during graft-versus-host-reactivity. The data presented here shows that Th9 cells: (1) can be polarized in the presence of rapamycin, (2) possess cytokine phenotype stability in vivo during allogeneic BMT; and (3) inhibit IFN-γ mediated graft-versus-host-reactivity.

The data presented here are the first to demonstrate that Th9 cell differentiation can proceed in the presence of rapamycin. The current finding extends previous observations on the relatively unbiased nature of CD4^+^ T cell differentiation in rapamycin [21]. The results presented here further confirm that the effect of rapamycin in blocking CD4^+^ T cell differentiation [12] can be overcome by using an APC free system along with polyclonal stimulation and adequate cytokine availability. Such expanded Th9.R cells were similar in cytokine phenotype and transcription factor profile to their non-rapa counterparts (Th9 cells). Also, in keeping with previous reports on Th9 cells [8] [2], we found that Th9.R cells were similar to Th2.R cells in terms of transcription factor expression and cytokine profile.

For the first time, we have demonstrated the phenotype stability of Th9.R cells in vivo during allogeneic BMT. Differentiation plasticity of T helper subsets during allogeneic BMT [22] [23] raises concerns with respect to the use of T helper cell therapy. Also, previous reports have suggested that Th9 cells are only transient IL-9 producers [24] and are prone to plasticity [25] in vivo during inflammation. In this context, it is noteworthy that we found that Th9.R cells inhibited type I cytokine production of allo-reactive T cells while maintaining their IL-9 producing capacity. Moreover, lineage stability was not exclusive to Th9.R cells but was also observed with respect to Th2.R cells. Taken together, these results indicate that expansion of T helper subsets in rapamycin may render lineage stability in vivo during inflammation, thereby enhancing the fidelity of defined T helper cell therapy.

This report is also the first to demonstrate an immune modulation function of Th9.R cell function during experimental allogeneic BMT. Specifically, we found that Th9.R cells inhibited IFN-γ driven alloreactivity with a potency similar to Th2.R cells. This finding indicates that it is possible to deliver cells with increased IL-9 secretion capacity without exacerbating alloreactivity. Further studies will be required to determine whether transfer of T cells with increased IL-9 secretion capacity do not increase GVHD or whether such cells might improve graft-versus-tumor-effects.

One might argue as others have previously asserted [2], that because the Th2.R and Th9.R cells had a similar in vitro phenotype and mediated similar in vivo inhibition of alloreactivity, these subsets are essentially the same T cell product and without substantial differential effect. However, contrary to this line of reasoning, we found that the Th2.R and Th9.R products had highly differential effects on donor T cell engraftment. Namely, donor Th9.R cells greatly inhibited donor CD8 engraftment during allogeneic BMT; Th2.R cells, on the other hand, did not inhibit donor CD8^+^ T cell engraftment. It is possible that the potent ability of Th9.R cells to limit CD8^+^ T cell engraftment may offer an improved balance to GVHD and GVT effects [9]. Similarly marked differential effects were also observed in terms of donor CD4^+^ T cell engraftment; that is, whereas Th2.R cell infusion increased donor CD4^+^ T cell engraftment, Th9.R cell infusion did not increase donor CD4^+^ T cell numbers post- BMT. Thus relative to the Th2.R population, Th9.R cells were equally efficient in terms of inhibiting IFN-γ driven alloreactivity and more potent in terms of limiting donor CD4^+^ and CD8^+^ T cell numbers post-BMT. Further studies are therefore warranted to determine the differential effects of Th2.R and Th9.R cell therapy in the modulation of transplant responses after allogeneic bone marrow transplantation.

## References

[1] Veldhoen M, Uyttenhove C, van Snick J, Helmby H, Westendorf A, et al (2008) Transforming growth factor-beta ‘reprograms’ the differentiation of T helper 2 cells and promotes an interleukin 9-producing subset. Nat Immunol 9: 1341–1346. 18931678 10.1038/ni.1659

[2] Gessner A, Blum H, Rollinghoff M (1993) Differential regulation of IL-9-expression after infection with Leishmania major in susceptible and resistant mice. Immunobiology 189: 419–435. 8125519 10.1016/S0171-2985(11)80414-6

[3] Dardalhon V, Awasthi A, Kwon H, Galileos G, Gao W, et al (2008) IL-4 inhibits TGF-beta-induced Foxp3^+^ T cells and, together with TGF-beta, generates IL-9^+^ IL-10^+^ Foxp3(-) effector T cells. Nat Immunol 9: 1347–1355. 18997793 10.1038/ni.1677 PMC2999006

[4] Khan WI, Richard M, Akiho H, Blennerhasset PA, Humphreys NE, et al (2003) Modulation of intestinal muscle contraction by interleukin-9 (IL-9) or IL-9 neutralization: correlation with worm expulsion in murine nematode infections. Infect Immun 71: 2430–2438. 12704113 10.1128/IAI.71.5.2430-2438.2003 PMC153239

[5] Cheng G, Arima M, Honda K, Hirata H, Eda F, et al (2002) Anti-interleukin-9 antibody treatment inhibits airway inflammation and hyperreactivity in mouse asthma model. Am J Respir Crit Care Med 166: 409–416. 12153980 10.1164/rccm.2105079

[6] Nicolaides NC, Holroyd KJ, Ewart SL, Eleff SM, Kiser MB, et al (1997) Interleukin 9: a candidate gene for asthma. Proc Natl Acad Sci U S A 94: 13175–13180. 9371819 10.1073/pnas.94.24.13175 PMC24282

[7] Wilhelm C, Hirota K, Stieglitz B, Van Snick J, Tolaini M, et al (2011) An IL-9 fate reporter demonstrates the induction of an innate IL-9 response in lung inflammation. Nat Immunol 12: 1071–1077. 21983833 10.1038/ni.2133 PMC3198843

[8] Poulin LF, Richard M, Le Moine A, Kiss R, McKenzie AN, et al (2003) Interleukin-9 promotes eosinophilic rejection of mouse heart allografts. Transplantation 76: 572–577. 12923446 10.1097/01.TP.0000071201.32424.D2

[9] Lu Y, Hong S, Li H, Park J, Hong B, et al (2012) Th9 cells promote antitumor immune responses in vivo. J Clin Invest 122: 4160–4171. 23064366 10.1172/JCI65459 PMC3484462

[10] Lu LF, Lind EF, Gondek DC, Bennett KA, Gleeson MW, et al (2006) Mast cells are essential intermediaries in regulatory T-cell tolerance. Nature 442: 997–1002. 16921386 10.1038/nature05010

[11] Sabers CJ, Martin MM, Brunn GJ, Williams JM, Dumont FJ, et al (1995) Isolation of a protein target of the FKBP12-rapamycin complex in mammalian cells. J Biol Chem 270: 815–822. 7822316 10.1074/jbc.270.2.815

[12] Battaglia M, Stabilini A, Migliavacca B, Horejs-Hoeck J, Kaupper T, et al (2006) Rapamycin promotes expansion of functional CD4^+^CD25^+^FOXP3^+^ regulatory T cells of both healthy subjects and type 1 diabetic patients. J Immunol 177: 8338–8347. 17142730 10.4049/jimmunol.177.12.8338

[13] Amarnath S, Costanzo CM, Mariotti J, Ullman JL, Telford WG, et al (2010) Regulatory T cells and human myeloid dendritic cells promote tolerance via programmed death ligand-1. PLoS Biol 8: e1000302. 20126379 10.1371/journal.pbio.1000302 PMC2814822

[14] Jung U, Foley JE, Erdmann AA, Toda Y, Borenstein T, et al (2006) Ex vivo rapamycin generates Th1/Tc1 or Th2/Tc2 Effector T cells with enhanced in vivo function and differential sensitivity to post-transplant rapamycin therapy. Biol Blood Marrow Transplant 12: 905–918. 16920556 10.1016/j.bbmt.2006.05.014

[15] Amarnath S, Flomerfelt FA, Costanzo CM, Foley JE, Mariotti J, et al (2010) Rapamycin generates anti-apoptotic human Th1/Tc1 cells via autophagy for induction of xenogeneic GVHD. Autophagy 6: 523–541. 20404486 10.4161/auto.6.4.11811 PMC3707503

[16] Amarnath S, Chen H, Foley JE, Costanzo CM, Sennesh JD, et al (2011) Host-based Th2 cell therapy for prolongation of cardiac allograft viability. PLoS One 6: e18885. 21559526 10.1371/journal.pone.0018885 PMC3084712

[17] Foley JE, Jung U, Miera A, Borenstein T, Mariotti J, et al (2005) Ex vivo rapamycin generates donor Th2 cells that potently inhibit graft-versus-host disease and graft-versus-tumor effects via an IL-4-dependent mechanism. J Immunol 175: 5732–5743. 16237064 10.4049/jimmunol.175.9.5732

[18] Mariotti J, Foley J, Ryan K, Buxhoeveden N, Kapoor V, et al (2008) Graft rejection as a Th1-type process amenable to regulation by donor Th2-type cells through an interleukin-4/STAT6 pathway. Blood 112: 4765–4775. 18625883 10.1182/blood-2008-05-154278 PMC2597142

[19] Mariotti J, Foley J, Jung U, Borenstein T, Kantardzic N, et al (2008) Ex vivo rapamycin generates apoptosis-resistant donor Th2 cells that persist in vivo and prevent hemopoietic stem cell graft rejection. J Immunol 180: 89–105. 18097008 10.4049/jimmunol.180.1.89

[20] Fowler DH, Mossoba ME, Steinberg SM, Halverson DC, Stroncek D, et al (2013) Phase 2 clinical trial of rapamycin-resistant donor CD4^+^ Th2/Th1 (T-Rapa) cells after low-intensity allogeneic hematopoietic cell transplantation. Blood 121: 2864–2874. 23426943 10.1182/blood-2012-08-446872 PMC3624934

[21] Amarnath S, Fowler DH (2012) Harnessing autophagy for adoptive T-cell therapy. Immunotherapy 4: 1–4. 22149992 10.2217/imt.11.144 PMC3249636

[22] Laurence A, Amarnath S, Mariotti J, Kim YC, Foley J, et al (2012) STAT3 transcription factor promotes instability of nTreg cells and limits generation of iTreg cells during acute murine graft-versus-host disease. Immunity 37: 209–222. 22921119 10.1016/j.immuni.2012.05.027 PMC3441059

[23] Amarnath S, Mangus CW, Wang JC, Wei F, He A, et al (2011) The PDL1-PD1 axis converts human TH1 cells into regulatory T cells. Sci Transl Med 3: 111ra120. 10.1126/scitranslmed.3003130 PMC3235958 22133721

[24] Tan C, Gery I (2012) The unique features of Th9 cells and their products. Crit Rev Immunol 32: 1–10. 22428852 10.1615/critrevimmunol.v32.i1.10 PMC3356583

[25] Tan C, Aziz MK, Lovaas JD, Vistica BP, Shi G, et al (2010) Antigen-specific Th9 cells exhibit uniqueness in their kinetics of cytokine production and short retention at the inflammatory site. J Immunol 185: 6795–6801. 20971929 10.4049/jimmunol.1001676 PMC2988091

